# Signal Oscillation Is Another Reason for Variability in Microarray-Based Gene Expression Quantification

**DOI:** 10.1371/journal.pone.0054753

**Published:** 2013-01-21

**Authors:** Raghvendra Singh

**Affiliations:** Department of Chemical Engineering, Indian Institute of Technology Kanpur, Kanpur, India; University of Westminster, United Kingdom

## Abstract

Microarrays have been widely used for various biological applications, such as, gene expression profiling, determination of SNPs, and disease profiling. However, quantification and analysis of microarray data have been a challenge. Previously, by taking into account translational and rotational diffusion of the target DNA, we have shown that the rate of hybridization depends on its size. Here, by mathematical modeling of surface diffusion of transcript, we show that the dynamics of hybridization on DNA microarray surface is inherently oscillatory and the amplitude of oscillation depends on fluid velocity. We found that high fluid velocity enhances the signal without affecting the background, and reduces the oscillation, thereby reducing likelihood of inter- and intra-experiment variability. We further show that a strong probe reduces dependence of signal-to-noise ratio on probe strength, decreasing inter-microarray variability. On the other hand, weaker probes are required for SNP detection. Therefore, we recommend high fluid velocity and strong probes for all microarray applications except determination of SNPs. For SNP detection, we recommend high fluid velocity with weak probe on the spot. We also recommend a surface with high adsorption and desorption rates of transcripts.

## Introduction

Although all cells of a multicellular organism share the same genes, their expression depends on the cell type and their microenvironment, which is responsible for difference in their phenotype and functionality [1]. Further, genes are switched on and off during various stages of development as well as in routine physiological functions such as cell-cycle [2], circadian clock [3], DNA repair [4], stress response [5], growth [6], and differentiation [7]. Besides normal variations in mRNA level, it has been found that gene expression switching is responsible for various pathological conditions including cancer [8]. For these reasons, DNA microarray is an important diagnostic tool, which has been used for determination of single nucleotide polymorphism [9], [10], gene expression profiling [11], profiling of diseases such as: diabetes [12], [13], HIV [14], [15], Alzheimer's disease [16], [17], Parkinson's disease [18], [19], Lyme disease [20], cancer [21]–[24], and malaria [25]. However, major challenge lies in understanding and analysis of thousands of noisy data points as well as in reducing the inter-experiment and inter-microarray variability [26]–[29]. The variability of microarray data has been attributed to biological variations, sample labeling error, technical variability, probe design, cross hybridization, low abundance of transcripts, and variability associated with control genes [28], [30]–[33]. To improve inter- and intra- microarray reproducibility, various design and statistical methods have been given, which have improved quantification and analysis of data obtained from these arrays [34]–[38]. Besides improving the data analysis, other aspects of DNA hybridization, such as its rate and efficiency, have been predicted using reaction-diffusion models. Previously, by taking into account rotational and translational diffusion, we have shown that the size of transcript plays an important role in the rate of hybridization [39]. Other models of surface DNA hybridization have also been given. For example, a model has been suggested that takes into account nonspecific adsorption of the target DNA followed by its 2D diffusion, which enhanced the hybridization efficiency [40]. Furthermore, Gadgil et. al. have predicted the minimum inter spot distance required to improve detection sensitivity using a reaction diffusion model [41].

Here, we modeled diffusion of a transcript in a microchannel over a spot and show that the hybridization process is inherently oscillatory. Since probe DNAs, the small complementary molecules (∼25 nucleotides) [42] immobilized on the surface, will be adsorbed inside and not accessible to transcripts in the solution, we hypothesize that solution-phase hybridization may be negligible in comparison to surface hybridization. Thus, we assume that the transcript adsorbs on the surface nonspecifically [40], then, diffuses on the site to find the probe DNA. However, desorption rate constant is very low at the spot, where it gets hybridized to the specific probe, than at other places on the surface. We further assume that hybridization reaction is much faster than the surface adsorption and diffusion of the target DNA and, thus, can be neglected. Our results show that signal oscillation is damped and increasing fluid velocity not only enhances its intensity but also reduces the amplitude of oscillation. We further found that although high fluid velocity reduces oscillation of the background signal as well, it does not increase its intensity. Thus, our study identifies fluid velocity as an important operational factor to reduce intra- and inter experiment variability. Furthermore, we show that for a strong probe the signal-to-noise ratio does not depend on the probe strength. Thus, immobilization of strong probes on the spot will improve inter-microarray reproducibility. In contrast, a weaker probe may be required for SNP detection.

## Mathematical Model

**Figure 1:**
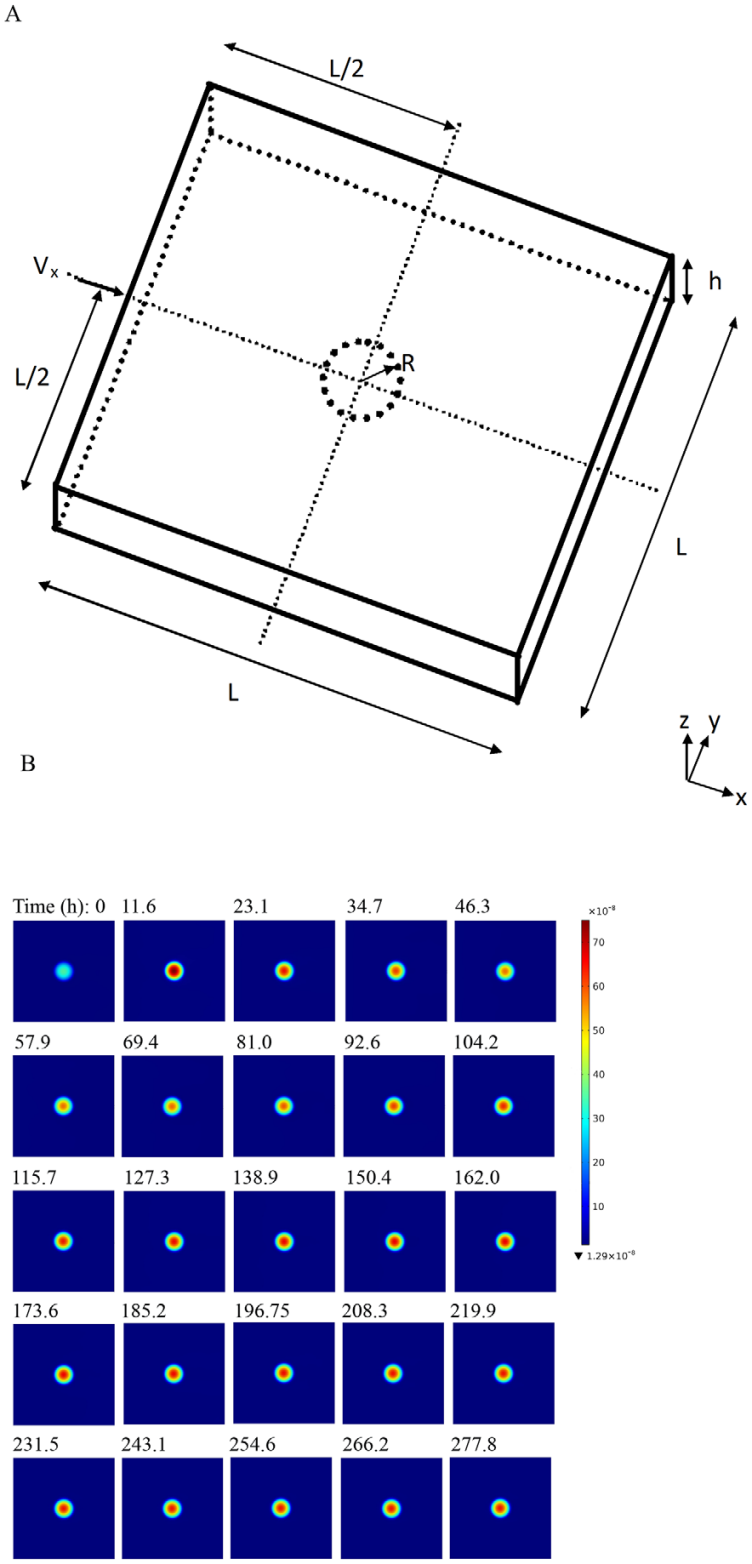
Schematic of hybridization channel and variation of hybridization intensity on the spot. (A) A square channel of side length L  = 100 μm and depth h  = 10 μm has been shown. At the mid-point of the channel, there is a spot of radius R = 10 μm on which target DNA hybridizes. The fluid flows in x-direction with velocity V_x._ (B) Pictures of spot intensity (xy-plane) at indicated time from COMSOL Multiphysics for a transcript concentration (P_0_) of 0.5 μM in the sample, adsorption rate constant (k_a_) of 0.1 m^3^/mol/s, specific desorption rate constant (k_d1_) of 1×10^−8^ s^−1^, concentration of adsorption sites (θ_0_) of 0.000104 mol/m^2^, surface diffusivity (D_s_) of 2×10^−13^ m^2^/s, solution diffusivity of 2×10^−11^ m^2^/s, nonspecific desorption rate constant (k_d2_) of 0.3 s^−1^ and the mid-point velocity (V_xmax_) of 5 mm/s. The color coded intensity is in mol/m^2^.

The diffusion of target DNA in the solution (Fig. 1A) is given by vector equation:
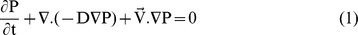
with initial condition: 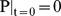 and boundary conditions:

at x  =  0 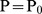


at x  =  L 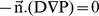


at y  =  0 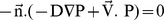


at y  =  L 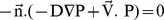


at z  =  h 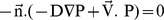


at z  =  0 and at the spot 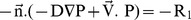


at z = 0 and excluding the spot 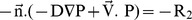


Diffusion of target DNA at the surface (at z = 0) is given by vector equation:

At the spot,
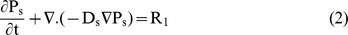
and at everywhere on the surface except the spot,



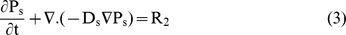
with initial condition: 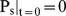 and boundary conditions: at z  =  0 and all edges of the surface,



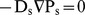
where,



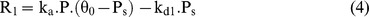


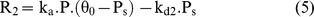



Velocity vector: 
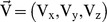


Due to channel size, the flow is considered laminar. Thus,
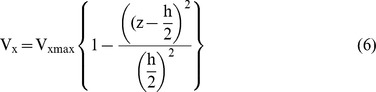



V_y_  = 0 and V_z_  = 0

Average concentration of the hybridized DNA has been calculated by integral:
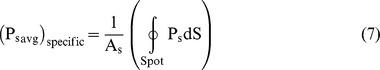
and average background intensity by



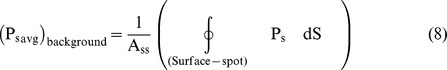



The signal to noise ratio at a particular hybridization time is calculated as:
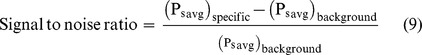
Where, P: concentration of the target DNA in hybridization channel (mol/m^3^); P_0_: concentration of the target DNA in sample (mol/m^3^); (P_savg_)_specific_: average concentration of hybridized target DNA on the spot (mol/m^2^); (P_savg_)_background_: average concentration of nonspecifically bound target DNA on the surface (mol/m^2^); D: solution diffusivity of target DNA (m^2^/s); 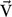: velocity vector (m/s); P_s_: concentration of DNA on the surface (mol/m^2^); D_s_: diffusivity of DNA on the surface (mol/m^2^); θ_0_: concentration of the adsorption sites (mol/m^2^); k_a_: adsorption rate constant at the surface (m^3^/s/mol); k_d1_: desorption rate constant at the spot (s^−1^); k_d2_: desorption rate constant at the surface excluding the spot (s^−1^); V_xmax_: mid-point fluid velocity in x-direction (m/s); A_s_: area of the spot (m^2^); A_ss_: area of the surface excluding the spot (m^2^); 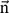: unit surface normal vector.

Values of range of surface adsorption, desorption rate constants, solution and surface diffusivities, representative concentration of target DNA in the sample, and DNA adsorption sites on the surface have been taken from literature for DNA hybridization [43], [44], [45]. The equations 1-9 have been solved using COMSOL Multiphysics 4.3a.

## Results

### Higher fluid velocity increases hybridization intensity and reduces signal oscillation

**Figure 2:**
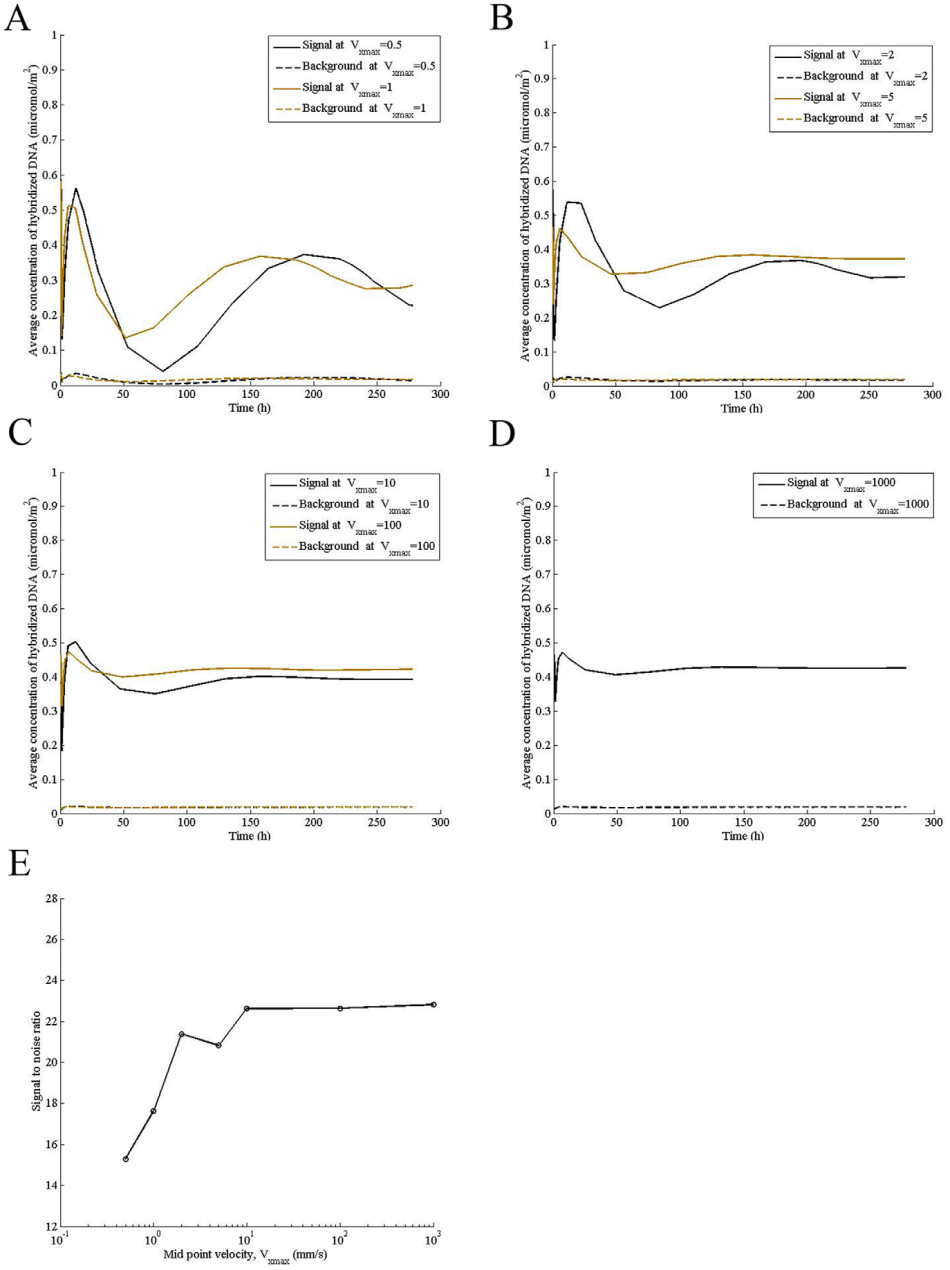
Higher fluid velocity increases hybridization intensity and reduces signal oscillation. Computations were performed at the indicated mid-point velocity (V_xmax_) for a transcript concentration (P_0_) of 0.5 μM in the sample, adsorption rate constant (k_a_) of 0.1 m^3^/mol/s, specific desorption rate constant (k_d1_) of 1×10^−8^ s^−1^, concentration of adsorption sites (θ_0_) of 0.000104 mol/m^2^, surface diffusivity (D_s_) of 2×10^−13^ m^2^/s, solution diffusivity of 2×10^−11^ m^2^/s, and nonspecific desorption rate constant (k_d2_) of 0.3 s^−1^. Signal-to-noise ratio was calculated at a hybridization time of 24 h.

Transcript in a microchannel (Fig. 1A) undergoes diffusion in solution and reaches the surface, where it is adsorbed nonspecifically. Following adsorption, it diffuses on the surface to reach the spot where probe molecules are immobilized and hybridizes. Concurrent to 2D diffusion and hybridization, DNA molecules desorb from the surface back to the solution, creating a feedback loop. The mathematical modeling of these reactions shows that concentration of DNA hybridized on the spot (Fig. 1B) as well as that of nonspecifically bound on the surface oscillates as hybridization progresses. The amplitude decreases, resulting in dampening and decreasing the variability of signal with time. For a fluid velocity of V_xmax_  = 0.5 mm/s, signal reaches a peak at t  = 12.4 h, then decreases sharply to reach close to the background level at t  = 80.9 h (Fig. 2A). Similar to the specific signal, damped oscillation of low amplitude is also seen in the background intensity. Increasing fluid velocity not only reduces the oscillation but also increases signal to noise ratio (Fig. 2A–E), improving microarray resolution. Although amplitude of oscillation continuously decreases with increase in fluid velocity, the signal to noise ratio saturates at V_xmax_ of 10 mm/s (Fig. 2E), suggesting that there may be an optimum velocity for DNA hybridization.

### Higher concentration of transcript does not improve signal resolution

**Figure 3:**
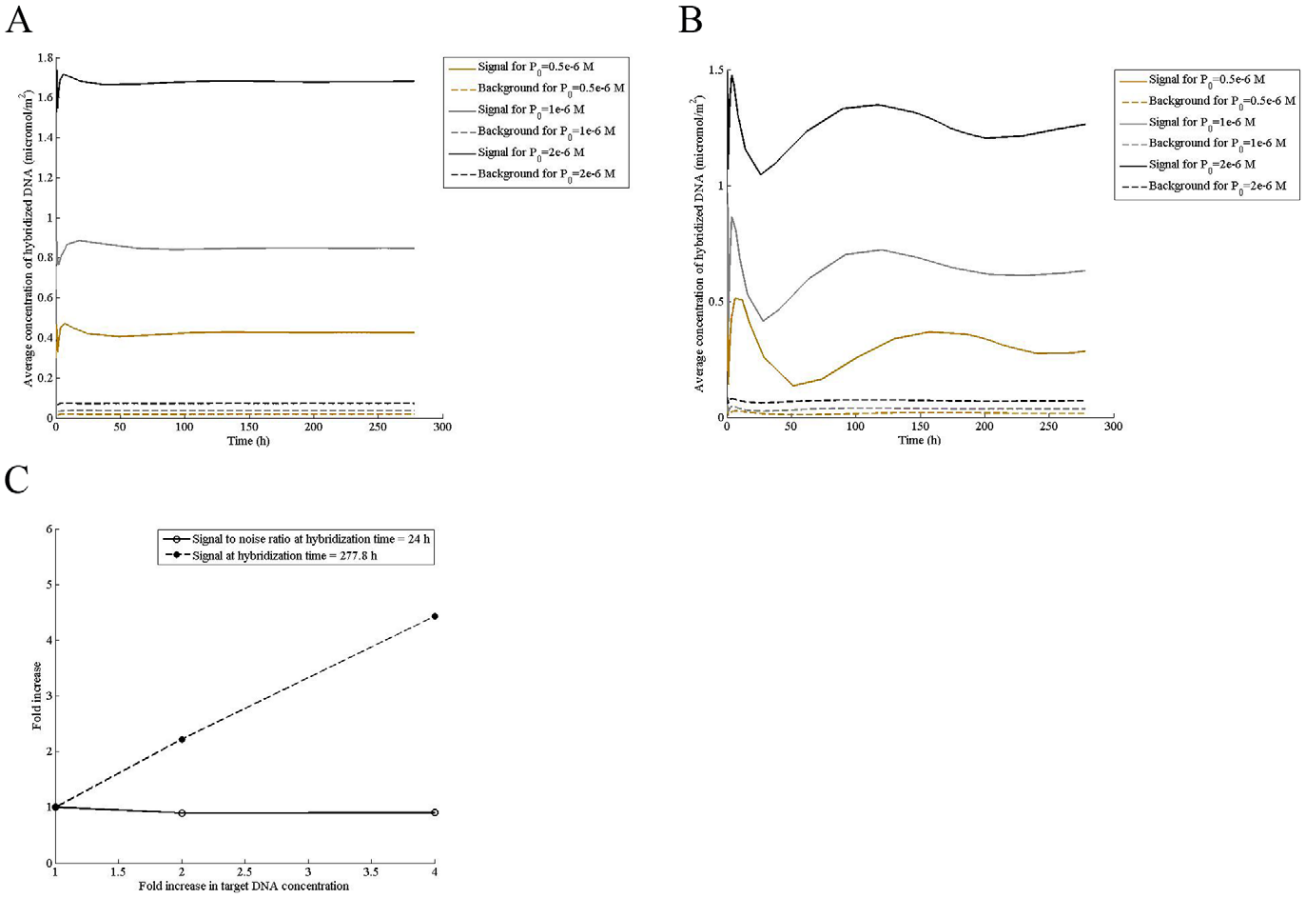
Higher concentration of transcript does not improve signal resolution. Computations were performed for indicated concentrations of the transcript in the sample for adsorption rate constant (k_a_) of 0.1 m^3^/mol/s, specific desorption rate constant (k_d1_) of 1×10^−8^ s^−1^, concentration of adsorption sites (θ_0_) of 0.000104 mol/m^2^, surface diffusivity (D_s_) of 2×10^−13^ m^2^/s, solution diffusivity of 2×10^−11^ m^2^/s, nonspecific desorption rate constant (k_d2_) of 0.3 s^−1^, and the mid-point velocity (V_xmax_) of (A) 1000 mm/s (B) 1 mm/s and (C) signal intensity and signal-to-noise ratio for the mid-point velocity (V_xmax_) of 1 mm/s at the indicated hybridization time.

Since quantification of scantily expressed genes has been a challenge, we studied the effect of transcript concentration on signal resolution. Although increasing transcript concentration increases the signal linearly, it has no effect on signal-to-noise ratio (Fig. 3C). Further, the oscillation is also not affected by the target DNA concentration (Fig. 3A, B), implying that sample concentration of the transcript does not improve signal resolution and reproducibility.

### Lower surface diffusivity of transcript enhances the signal while it simultaneously increases the amplitude of oscillation

**Figure 4:**
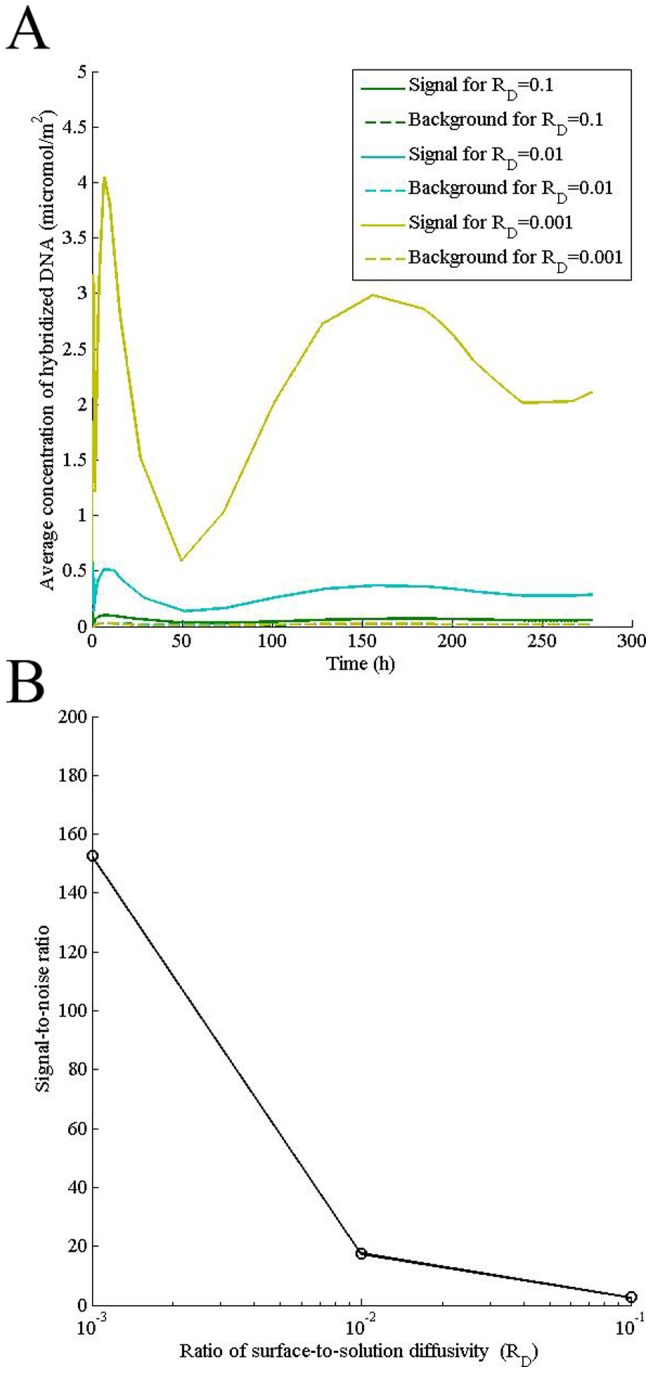
Lower surface diffusivity of transcript enhances the signal while it simultaneously increases the amplitude of oscillation. Computations were performed for indicated ratio of surface to solution diffusivity (R_D_) for a transcript concentration (P_0_) of 0.5 μM in the sample, adsorption rate constant (k_a_) of 0.1 m^3^/mol/s, specific desorption rate constant (k_d1_) of 1×10^−8^ s^−1^, concentration of adsorption sites (θ_0_) of 0.000104 mol/m^2^, solution diffusivity of 2×10^−11^ m^2^/s, nonspecific desorption rate constant (k_d2_) of 0.3 s^−1^, and the mid-point velocity (V_xmax_) of 1 mm/s. Signal-to-noise ratio was calculated at a hybridization time of 24 h.

DNA microarrays have been traditionally used to compare gene expression under different treatment conditions although their application in quantifying absolute concentration of different transcripts, including splice variants, is equally significant, specifically in disease profiling, and has been increasing progressively. Since one of the major reactions in hybridization process is surface diffusion of the transcript, its 2D diffusivity, which decreases as the transcript length increases, is an important parameter that governs the signal intensity. We found that decreasing surface diffusivity of the transcript for a fixed solution diffusivity increases both signal-to-noise ratio (Fig. 4B) and amplitude of oscillation (Fig. 4A), suggesting that although signal resolution becomes better at any time-point, its time-variability increases with increase in transcript length, which may lower the inter-experiment reproducibility.

### A strong probe lowers the inter-microarray variability while a weak probe may be required for SNP detection

**Figure 5:**
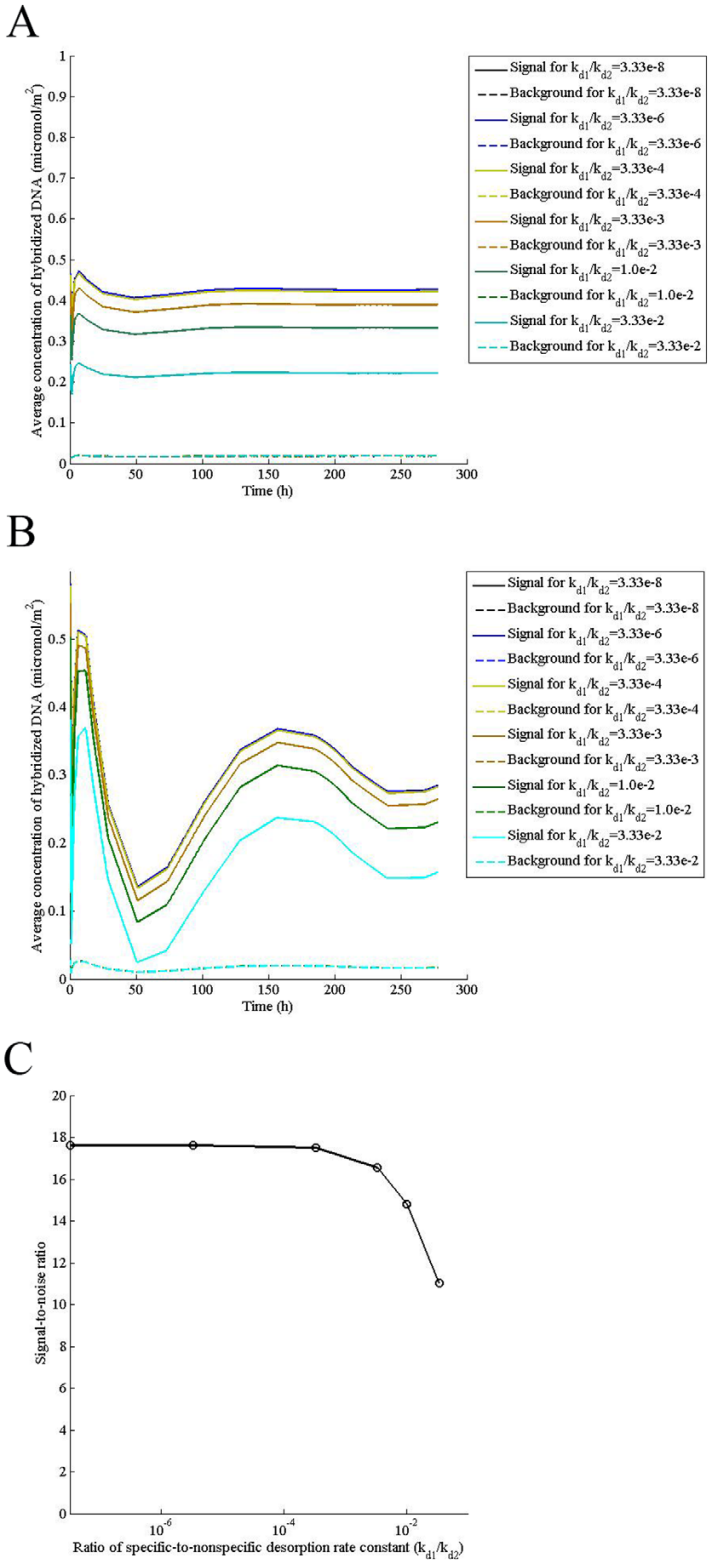
A strong probe lowers the inter-microarray variability while a weak probe may be required for SNP detection. Computations were performed for indicated ratios of specific/nonspecific desorption rate constants (k_d1_/k_d2_) for a transcript concentration (P_0_) of 0.5 μM, the adsorption rate constant (k_a_) of 0.1 m^3^/mol/s, nonspecific desorption rate constant (k_d2_) of 0.3 s^−1^, concentration of adsorption sites (θ_0_) of 0.000104 mol/m^2^, solution diffusivity of 2×10^−11^ m^2^/s, surface diffusivity (D_s_) of 2×10^−13^ m^2^/s, and the mid-point velocity (V_xmax_) of (A) 1000 mm/s (B) 1 mm/s and (C) Signal-to-noise ratio was calculated for V_xmax_  = 1 mm/s at a hybridization time of 24 h.

Probe selection, including its specificity and affinity to the transcript, constitutes an integral part of microarray design. Since specific desorption rate constant depends on probe length and its GC content, we studied its effect on hybridization. When specific desorption rate constant is low due to a strong probe immobilized on the spot, increasing it by four orders of magnitude has no effect on either the dynamics of signal or signal-to-noise ratio (Fig. 5A, B, C), implying that selection of a strong probe causes signal to be independent of probe design, improving inter-microarray reproducibility. On the other hand, if a weak probe, which means high specific desorption rate constant, has been immobilized on the surface, increasing the desorption rate constant decreases signal to noise ratio sharply (Fig. 5C), suggesting that a weak probe may increase inter-microarray variability although it may be preferred for SNP detection.

### Specific and nonspecific equilibrium constants have opposite effects on signal resolution while both increase the oscillation

**Figure 6:**
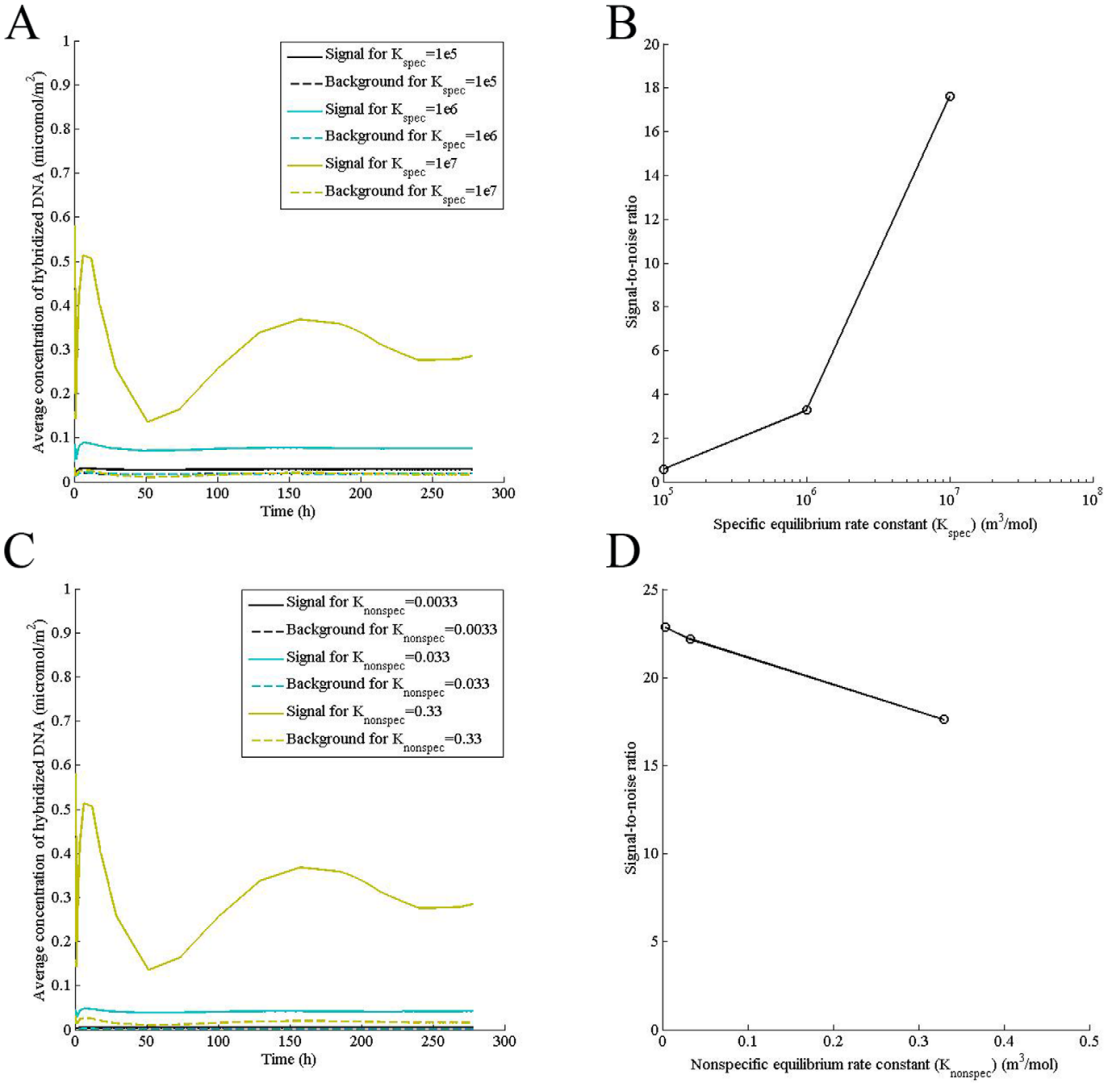
Specific and nonspecific equilibrium constants have opposite effects on signal resolution while both increase the oscillation. Computations were performed for indicated specific (K_spec_, m^3^/mol) and nonspecific (K_nonspec_, m^3^/mol) equilibrium constants for a transcript concentration (P_0_) of 0.5 μM, concentration of adsorption sites (θ_0_) of 0.000104 mol/m^2^, solution diffusivity of 2×10^−11^, surface diffusivity (D_s_) of 2×10^−13^ m^2^/s, and the mid-point velocity (V_xmax_) of 1 mm/s and (A) for a specific desorption rate constant (k_d1_) of 1×10^−8^ s^−1^ (B) Signal-to-noise ratio was calculated at a hybridization time of 24 h and for a specific desorption rate constant (k_d1_) of 1×10^−8^ s^−1^ (C) for a nonspecific desorption rate constant (k_d2_) of 0.3 s^−1^(D) Signal-to-noise ratio was calculated at a hybridization time of 24 h and for a nonspecific desorption rate constant (k_d2_) of 0.3 s^−1^.

Adsorption and desorption rate constants are measures of how fast a transcript adsorbs and releases from a surface, respectively, while equilibrium constant is a measure of amount of surface adsorbed DNA, which remains in equilibrium with that in the solution. Thus, specific equilibrium constant is a measure of transcript hybridized on the spot while the nonspecific equilibrium constant is a measure of transcript adsorbed nonspecifically on the surface. Along with probe selection, surface characteristics play an important role in the hybridization process. We found that increasing the specific equilibrium constant while keeping the nonspecific equilibrium constant fixed increases signal intensity without affecting the background, thus, enhancing the resolution (Fig. 6A, B). In contrast, increasing nonspecific equilibrium constant increases both the signal and the background, decreasing signal-to-noise ratio (Fig. 6C, D). Further, increasing either the specific or nonspecific equilibrium constant increases amplitude of oscillation, implying that higher equilibrium constants may increase inter-experiment variability (Fig. 6A, C).

## Discussion

We found that dynamics of surface DNA hybridization is oscillatory, which is a characteristics of a feedback mechanism [46], [47]. In this case, feedback mechanism is 2D diffusion and desorption of transcript from surface back to the solution. Slower the surface diffusion and/or the desorption rate, higher the delay in feedback, and higher the amplitude of oscillation. Increasing fluid velocity causes mixing in the solution, increasing convective mass transfer, thereby, enhancing the signal and reducing the amplitude of oscillation. Although fluid velocity causes mixing effect, the overall hybridization rate is governed by slower 2D diffusion and adsorption processes. Thus, after a certain velocity signal-to-noise ratio saturates. While high signal-to-noise ratio is preferred for detection in a single experiment, signal oscillation may be responsible for inter-experiment and inter-microarray variability, along with other reasons [48]. Thus, hybridization at higher fluid velocity may increase sensitivity and reproducibility of microarray, as have been found experimentally [49], [50]. In agreement with our study, time series data obtained using microarray showed genome wide oscillation [51], [52] although it is not clear whether a part of it may be attributed to that inherent with microarrays.

Increasing transcript concentration increases the signal regardless of the fluid velocity. Since background signal also increases, transcript concentration has no effect on signal to noise ratio. Thus, a minimum concentration of target DNA is required so that the signal can be detected by available methods.

Lower surface diffusivity of transcript improves the signal as it reduces the likelihood of DNA diffusing out of the spot. However, it also increases the amplitude of oscillation since the feedback mechanism from the surface becomes even slower. Thus, a surface on which diffusivity of transcript is low increases the signal to noise ratio, improving detection in a single experiment. On the other hand, due to higher oscillation, inter-experiment and inter-microarray variability may increase.

Desorption rate constant of transcript at the spot depends on probe design. A strong probe of longer length and/or high GC content will decrease the specific desorption rate constant. The study shows that for such probes the signal-to-noise ratio does not depend on the probe design, thereby, reducing the inter-microarray variability. On the other hand, for SNP detection, it is desired that increasing desorption rate constant due to single nucleotide mismatch lowers the signal to noise ratio, differentiating the transcripts with polymorphism. Therefore, for SNP detection, a probe of shorter length and lower GC content may be preferred in agreement with recent experimental finding [53].

Increasing surface adsorption rate constant while keeping the nonspecific equilibrium constant unchanged improves the signal-to-noise ratio since it increases the specific binding at the spot without affecting the background. On the other hand, increasing the nonspecific equilibrium constant while keeping the specific equilibrium constant unchanged decreases the signal-to-noise ratio due to increased background intensity. Since adsorption and nonspecific desorption are surface properties, it is desirable to have a surface that has high adsorption and desorption rates of DNA so that specific binding is high while background is low, improving resolution in a specific experiment. However, higher adsorption rate aggravates the delay in feedback by the 2D diffusion followed by desorption, increasing the signal oscillation. Thus, low adsorption rate on the surface will improve inter-experiment and inter-microarray reproducibility.

In summary, a number of design and operational implications can be drawn from the study (Table 1). First, vigorous and uniform mixing, either in a microfluidic device or through agitation, is desired. Second, although sensitivity of microarray continues to be a major challenge [28], transcript concentration beyond the sensitivity threshold may not reduce microarray variability. Further, strong probe will improve signal-to-noise ratio and inter-microarray reproducibility. Although higher GC content is preferred, it may be primarily controlled by annealing temperature requirement of the probes. On the other hand, probes of longer length can be used although their effect on signal specificity needs further investigation [28]. In the range of target DNA concentration considered in the study, signal intensity at a given time increases linearly with increase in transcript concentration. Thus, microarray can be calibrated to measure the absolute concentration. However due to oscillation, either the hybridization time should be fixed or the experiment should be performed under vigorous mixing for absolute concentration measurement. Furthermore, lower surface-to-solution diffusivity ratio of target DNA improves signal-to-noise ratio. Since 2D diffusivity depends on polymer length much strongly than the solution diffusivity [54], transcripts of longer length may improve resolution. Although for measurement of ratios of the same transcript under different treatment conditions its length may not be important, for comparison of amounts of different transcripts including splice variants the signal intensity should be corrected for difference in transcript length.

**Table 1:** Effect of operational and design factors on microarray variability.

Factor	Effect		Implication			
	Signal oscillation	Signal-to- noise ratio	Intra-experiment variability	Inter-experiment variability	Inter-microarray variability	SNP detection
Higher fluid velocity	Lower	Higher	Lower	Lower	Lower	Preferred
Higher concentration of transcript	No effect	No effect	No effect	No effect	No effect	No effect
Higher surface to solution diffusivity ratio	Lower	Lower	Higher	May decrease	May decrease	Not preferred
Higher ratio of specific to nonspecific desorption rate constant	No effect	Lower	Higher	No effect	Higher	Preferred
Higher specific equilibrium constant	Higher	Higher	Lower	Higher	Higher	Preferred
Higher nonspecific equilibrium constant	Higher	Lower	Higher	Higher	Higher	Not preferred
